# Assessing the Suitability of ChatGPT and DeepSeekAI for Parent's Education on Common Pediatric Respiratory Diseases

**DOI:** 10.7759/cureus.92434

**Published:** 2025-09-16

**Authors:** Anna Jijo, Maria Jijo, Swetha Manoj, Anitha Veerachamy

**Affiliations:** 1 Pediatrics, St. John's Medical College, Bangalore, IND; 2 Pediatrics, Apollo Institute of Medical Sciences and Research, Hyderabad, IND; 3 Pediatrics and Neonatology, Government Medical College Kannur, Kannur, IND; 4 Pediatrics, Meenakshi Mission Hospital &amp; Research Centre, Madurai, IND

**Keywords:** artificial intelligence, chatgpt, deepseek, deepseekai, education, paediatric respiratory diseases, pediatric respiratory diseases

## Abstract

Introduction

Parental education on pediatric respiratory illnesses is essential to ensure timely care for children. With the increasing use of artificial intelligence (AI) in health communication, tools such as ChatGPT and DeepSeekAI offer potential for generating accessible and reliable educational material.

Methodology

A cross-sectional analysis was conducted using AI-generated educational content. Each response was assessed for word and sentence count, average words per sentence, syllables per word, readability (Flesch Reading Ease Score and Grade Level), similarity percentage (QuillBot), and reliability (modified Discern Score). Statistical analysis was performed using independent sample t-tests, with p<0.05 considered significant.

Results

DeepSeekAI responses were longer (mean word count: 422 vs. 333.75) and included more sentences. However, no statistically significant differences were found in any variable, including readability (Grade Level: 9.22 vs. 9.30; Ease Score: 41.17 vs. 40.97) and reliability (2.25 vs. 2.00). Similarity scores were also comparable between the two tools (36.02 vs 32.1).

Conclusion

Both ChatGPT and DeepSeekAI generated parent education materials of similar quality, readability, and reliability. The findings of this study suggest that either AI tools can be utilized for developing parental educational content for common pediatric respiratory conditions.

## Introduction

Pediatric respiratory illnesses such as croup, bronchiolitis, pneumonia, and pertussis remain major causes of morbidity and healthcare utilization in children under five years of age, accounting for a substantial proportion of pediatric emergency visits and hospitalizations worldwide [1,2]. Croup, most often caused by parainfluenza viruses, presents with stridor and a characteristic barking cough, with peak incidence during the fall and winter months. Bronchiolitis, typically linked to respiratory syncytial virus (RSV), continues to be the leading cause of hospitalization in infants under 12 months of age, particularly during the winter season [1]. Pneumonia, whether viral or bacterial, is a major contributor to respiratory distress and inpatient care, especially in children with comorbid conditions [2]. A recent study analyzing pediatric hospital admissions for acute respiratory diseases reported that out of 52,839 hospitalizations, 30.3% were due to bronchiolitis, 27.2% due to pneumonia, 15.1% due to influenza-like illness, and 5.7% due to croup, highlighting that more than 60% of these admissions were related to respiratory conditions [3].

Parental awareness and early recognition of these conditions are critical for timely intervention, effective home management, and reducing avoidable admissions. However, low health literacy remains a substantial barrier, and studies suggest that nearly one-third of caregivers worldwide have limited health literacy, with even higher prevalence reported in underserved populations [4]. Importantly, low health literacy often overlaps with low digital or artificial intelligence (AI) literacy, meaning that caregivers with limited medical knowledge may also face difficulties in interpreting AI-generated educational material. This dual gap raises the question of whether AI platforms can genuinely bridge inequities by simplifying access to information or whether they risk compounding existing barriers if not carefully designed and validated.

With recent advances in large language models, AI has emerged as a promising tool to support public health education. ChatGPT (OpenAI) and DeepSeekAI are two prominent conversational platforms that provide personalized, real-time responses to user queries. These tools can help caregivers better understand symptoms, home care strategies, and red flag signs requiring urgent attention. AI-based platforms are highly scalable and accessible, making them potentially valuable in low-resource settings. At the same time, challenges such as variable accuracy, lack of context-specific guidance, and the possibility of misinformation emphasize the importance of ongoing validation and integration with evidence-based pediatric guidelines [5,6].

AI-driven patient education has specific utility in the context of pediatric respiratory conditions. For croup, AI platforms can help caregivers distinguish between mild cases managed at home and severe presentations requiring emergency care, especially through the recognition of signs such as stridor at rest. In bronchiolitis, ChatGPT-AI and DeepSeekAI can support parental decision-making regarding hydration, symptom monitoring, and when to escalate care. For pneumonia, these tools may help identify symptoms suggestive of bacterial infection, such as high fever and respiratory distress, guiding families toward timely medical evaluation. In the case of pertussis, AI platforms can reinforce the importance of immunization, early detection of paroxysmal coughing episodes, and the urgency of seeking care for infants at risk of apnea or severe complications. When appropriately designed and validated, AI technologies can complement traditional health systems by enhancing parental counseling, supporting preventive care, and improving child health outcomes.

Aims and objectives

This study aims to compare the readability, similarity, and reliability of parent education materials generated by ChatGPT and DeepSeekAI for four common pediatric respiratory conditions: croup, bronchiolitis, pertussis, and pneumonia. It aims to draw a comparison based on readability and ease of understanding.

## Materials and methods

A cross-sectional original research study was conducted over one week, from March 1 to March 7, 2025. Since no human participants were involved, the study did not require ethical clearance from an institutional review board. The primary objective was to evaluate the readability, originality, and reliability of AI-generated patient education materials on common pediatric respiratory conditions. For this purpose, two advanced AI models were selected: ChatGPT (GPT-4, OpenAI) and DeepSeekAI (DeepSeek-V2). Both tools were tasked with generating educational brochures based on standardized prompts for four pediatric conditions: croup, bronchiolitis, pertussis, and pneumonia. The prompts used were as follows: “Write a patient education guide on [condition]”. All responses were generated and collected in Microsoft Word format between March 1 and March 7, 2025.

To assess the quality of the generated texts, a multi-step evaluation was conducted. First, readability was evaluated using the Flesch-Kincaid Calculator, which analyzed total word count, sentence count, and readability score based on ease of understanding. Second, originality and similarity were assessed using the QuillBot Plagiarism Checker, which scanned each document for overlap with existing content. Third, reliability and quality were graded using a modified DISCERN tool, which is a validated instrument developed to judge the quality of written consumer health information. The DISCERN tool includes five core questions: Are the aims clear and achieved? Are reliable sources of information used? Is the information presented both balanced and unbiased? Are additional sources of information listed for patient reference? Are areas of uncertainty mentioned? Each response was independently rated using this scale to ensure content clarity, trustworthiness, and patient-centeredness [7].

All compiled data were exported to Microsoft Excel (Microsoft Corp., Redmond, WA) and analyzed using R version 4.3.2 (R Foundation for Statistical Computing, Vienna, Austria). Descriptive statistics were used to summarize readability and reliability scores. Comparisons between ChatGPT and DeepSeekAI outputs were made using an unpaired t-test, with a p-value <0.05 considered statistically significant. Additionally, the relationship between readability and reliability was evaluated using Pearson’s coefficient of correlation. The comprehensive analysis allowed for an objective comparison of AI-generated health education tools in a pediatric context.

 This article was previously posted to the Authorea preprint server on July 14, 2025.

## Results

**Figure 1:**
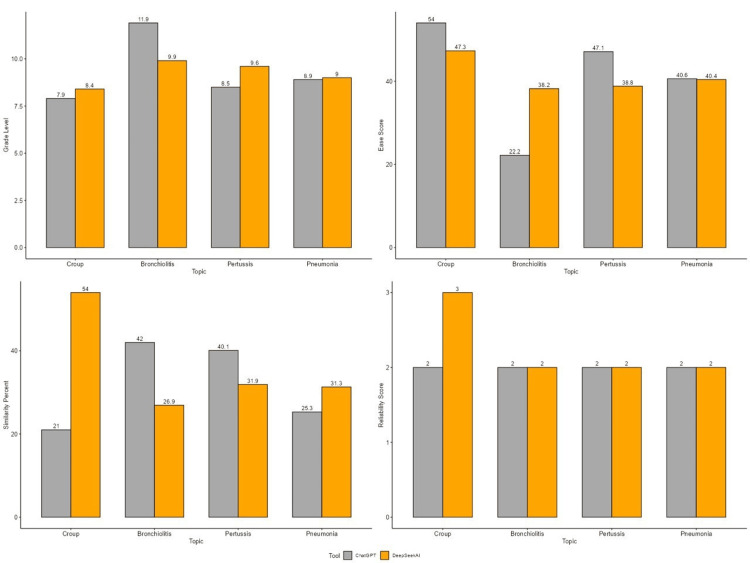
Graphical representation of comparison between grade level, ease score, similarity percent, and reliability score for the patient education guide generated by ChatGPT and DeepSeekAI.

ChatGPT and DeepSeekAI were used to generate brochures on patient education regarding croup, bronchiolitis, pertussis, and pneumonia. Figure 1 displays a comparison between ChatGPT and DeepSeekAI on four patient education topics across four key features: grade level, ease score, similarity percent, and reliability score. Table 1 summarizes the characteristics of responses generated by ChatGPT and DeepSeekAI. Across all variables, including word count, sentence count, readability indices, similarity percentage, and reliability scores, no statistically significant differences were observed (all p > 0.05). Both AI tools produced materials at approximately a ninth-grade reading level, with comparable readability (Ease Score ~41) and reliability (DISCERN ~2). These findings suggest that, from an educational standpoint, both platforms generate content of similar complexity and perceived trustworthiness, making them equally suitable for parental education.

**Table 1:** Characteristics of responses generated by ChatGPT and DeepSeekAI in regard to number of words, sentences, grade level, ease score, similarity percentage, and reliability score. ^+^t-test. P-values < 0.05 are considered statistically significant.

	ChatGPT		DeepSeekAI		P-value^+^
	Mean	Standard Deviation	Mean	Standard Deviation	
Words	333.75	133.15	422.00	86.37	0.309
Sentences	48.50	22.84	62.50	17.14	0.365
Average words per sentence	7.12	1.44	6.92	0.94	0.823
Average syllables per word	1.87	0.17	1.87	0.05	1.000
Grade level	9.30	1.78	9.22	0.67	0.940
Ease score	40.97	13.66	41.17	4.18	0.979
Similarity %	32.10	10.51	36.02	12.19	0.643
Reliability score	2.00	0.00	2.25	0.50	0.391

In terms of word count, ChatGPT produced a mean of 333.75 (±133.15), while DeepSeek AI produced a slightly higher mean of 422.00 words (±86.37). However, the p-value of 0.309 suggests that the values are not statistically significant and that the responses generated are of comparable length overall.

In terms of sentence count, ChatGPT produced a mean of 48.50 sentences (±22.84), while DeepSeek AI produced a mean of 62.50 sentences (±17.14). The p-value of 0.365 indicates that the values are not statistically significant and that the AI tools are able to present information in a similarly segmented way.

In terms of average words per sentence, Chat GPT produced a mean of 7.12, and, similarly, DeepSeekAI produced a mean of 6.92. With only a slight difference between the two, a p-value of 0.823 indicates the values to not be of statistical significance. Both the models have favored similar sentence construction.

For the average syllables per word, which indicates the complexity of the vocabulary used, ChatGPT and DeepSeekAI both produced a mean of 1.87 syllables per word. The p-value of 1.000 indicates that there is absolutely no difference in this parameter.

For the overall grade level, ChatGPT and DeepSeekAI produced content at a mean level of 9.30 and 9.22, respectively (p = 0.940), indicating no statistically significant difference. Both outputs were written at approximately the ninth-grade level, which is above the recommended sixth- to eighth-grade reading level for patient-facing educational material. This suggests that while both tools generate comparable text, their readability may still pose challenges for caregivers with limited health literacy.

Ease scores were nearly identical (ChatGPT: 40.97, DeepSeekAI: 41.17; p = 0.979). On the Flesch Reading Ease scale (0 = very difficult, 100 = very easy), scores around 40 are typically comparable to academic texts or technical pamphlets, indicating that both AI models produced brochures that would be considered fairly difficult for a general audience.

Similarity percentages were also comparable (ChatGPT: 32.10%, DeepSeekAI: 36.02%; p = 0.643). In plagiarism detection benchmarks, similarity values below ~40% are generally considered acceptable for educational content, particularly when they reflect overlap with commonly used phrases in medical communication. ChatGPT consistently tended to produce lower similarity percentages, suggesting relatively higher originality in its outputs.

Reliability scores, assessed using the modified DISCERN tool, were 2.00 for ChatGPT and 2.25 for DeepSeekAI (p = 0.391), again indicating no significant differences. Both scores fall within the range, suggesting medically acceptable, though not comprehensive, information.

While topic-specific sub-analyses (croup, bronchiolitis, pertussis, pneumonia) revealed some variations, such as ChatGPT showing higher readability scores in croup and bronchiolitis, none of these differences reached statistical significance. To improve readability of the results, detailed sub-analysis values have been moved to a supplementary table.

Take-home point

No statistically significant differences were identified across readability, similarity, or reliability domains, suggesting that both AI tools perform comparably in generating parent education materials for pediatric respiratory illnesses.

## Discussion

A cross-sectional study conducted to compare responses generated by the AI tools ChatGPT and DeepSeekAI for brochures on patient education for common pediatric respiratory conditions such as croup, bronchiolitis, pertussis, and pneumonia revealed that there is no statistically significant difference in grade level, ease score, similarity percent, and reliability score between the two AI tools. These findings suggest that both platforms can be considered comparable tools for generating patient education content, with potential applications for clinicians and healthcare systems seeking scalable, low-cost solutions for parental counseling.

Content produced by AI for patient education provides an opportunity for those in resource-limited areas to gain access to health information. It allows patients to make informed and timely decisions on their health [8]. Despite their widespread use, traditional patient education methods are frequently limited by issues such as inadequate information retention, communication barriers due to language differences, and anxiety on the part of both patients and healthcare providers. Beyond facilitating informed decision-making, it also promotes greater patient adherence and leads to more favorable clinical outcomes [9]. To ensure that information provided in the materials is easy to read and retain by the general public, care should be taken to keep them simplified and concise [10]. In this study, there is no statistically significant difference between the average words, sentences, words per sentence, and syllables per sentence between the two AI tools. The readability of easy-to-read health brochures is recommended as at or below sixth- to eighth-grade level [11]. In this study, the average ease score of the content produced by ChatGPT was 40.97 and that of DeepSeekAI was 41.17, which indicates college-grade level. Neither tool achieved the recommended readability target, suggesting that further refinement is needed before AI outputs can be widely adopted for patient-facing education.

Several studies have been conducted to assess the role of AI in patient education on health conditions, including a study to understand AI’s role in endometriosis patient education. The findings indicated that the responses were accurate with a varying degree of sufficiency [12]. A randomized controlled trial comparing an AI-enabled patient decision aid with education alone in patients with advanced knee osteoarthritis demonstrated that the AI tool significantly improved decision quality, satisfaction, and shared decision-making [13]. These findings highlight the potential of AI and also point to the importance of addressing risks such as the digital divide, AI literacy, and misinformation, which could limit equitable benefits.

AI tools such as ChatGPT are trained using massive text datasets in multiple languages, which is then used to provide adequate responses to the input that is given. This can lead to outputs that resemble existing text, sometimes resulting in overlap with published material [14]. Such overlap may be unintentional, reflecting the reuse of common medical phrases [15,16]. In this study, the average similarity percent using ChatGPT was 32.10 and that using DeepSeekAI was 36.02, both within acceptable ranges for patient education materials. A study on plagiarism in medical writing highlights that duplication can be unintentional and that appropriate paraphrasing and citation are important safeguards [17,18].

The DISCERN tool is an instrument developed to assess the reliability of health information, primarily designed for use by researchers and clinicians [19]. Patients themselves may not routinely apply it in real-world contexts, though the principles underlying the tool reflect broader aspects of information trustworthiness. Banasiak and Meadows-Oliver applied the modified DISCERN score to asthma websites and found that HONcode-certified sites had higher reliability [20]. In our study, the average DISCERN scores were 2 for ChatGPT and 2.25 for DeepSeekAI, with no statistically significant difference, suggesting that both models generated medically acceptable, though not comprehensive, information. Similar studies comparing AI chatbots for hypertension and breastfeeding queries also concluded that while reliability was reasonable, accuracy and usability remained concerns, indicating the need for ongoing monitoring and integration with healthcare oversight [9,21].

Limitations

This study is limited by the evaluation of only two AI tools and four diseases. Future research must include a broader range of AI tools and medical conditions to assess the suitability of AI for patient education. Additionally, the version of ChatGPT used in this study is not the most recent, and it cannot be assumed that it provides the latest medical information. Given the rapid advancements in medicine and AI, model evolution may affect reproducibility of findings. Furthermore, readability and reliability scores do not capture the accuracy of medical content, which is an equally important dimension for patient education.

## Conclusions

This study highlights that there is no statistically significant difference in the average grade level, ease score, similarity percent, and reliability score for parent education guides generated by ChatGPT and DeepSeekAI on croup, bronchiolitis, pertussis, and pneumonia in the pediatric population. Both tools produced content of comparable complexity and reliability, though neither met the recommended readability level for patient-facing materials. Further research must be undertaken to include other AI tools and a variety of medical conditions, particularly those of current public health relevance such as COVID-19, diabetes, and hypertension. Mechanisms, such as integration with health systems, oversight by medical professionals, and open access repositories, will be essential to ensure accuracy and equitable access. Ultimately, AI-generated medical content must be accessible, up-to-date, and verifiable to meaningfully enhance patient education.

## References

[1] Hall CB, Weinberg GA, Blumkin AK (2013). Respiratory syncytial virus-associated hospitalizations among children less than 24 months of age. Pediatrics.

[2] Jain S, Williams DJ, Arnold SR (2015). Community-acquired pneumonia requiring hospitalization among U.S. children. N Engl J Med.

[3] Lukac CD, Simms B, Kwong GP, Holodinsky JK, Johnson DW, Kellner JD (2025). Hospitalizations for all-cause pediatric acute respiratory diseases in Alberta, Canada, before, during, and after the COVID-19 pandemic: a population-level retrospective cohort study from 2010 to 2024. Lancet Reg Health Am.

[4] DeWalt DA, Broucksou KA, Hawk V, Brach C, Hink A, Rudd R, Callahan L (2011). Developing and testing the health literacy universal precautions toolkit. Nurs Outlook.

[5] Blease C, Kaptchuk TJ, Bernstein MH, Mandl KD, Halamka JD, DesRoches CM (2019). Artificial intelligence and the future of primary care: Exploratory qualitative study of UK general practitioners’ views. J Med Internet Res.

[6] Topol EJ (2019). High-performance medicine: the convergence of human and artificial intelligence. Nat Med.

[7] Charnock D, Shepperd S, Needham G, Gann R (1999). DISCERN: an instrument for judging the quality of written consumer health information on treatment choices. J Epidemiol Community Health.

[8] Ihsan MZ, Apriatama D, Pithriani Pithriani, Amalia R (2025). AI-assisted patient education: challenges and solutions in pediatric kidney transplantation. Patient Educ Couns.

[9] Vinufrancis A, Al Hussein H, Patel HV, Nizami A, Singh A, Nunez B, Abdel-Aal AM (2024). Assessing the quality and reliability of AI-generated responses to common hypertension queries. Cureus.

[10] Bothun LS, Feeder SE, Poland GA (2022). Readability of COVID-19 vaccine information for the general public. Vaccine.

[11] Thorat V, Rao P, Joshi N, Talreja P, Shetty AR (2024). Role of artificial intelligence (AI) in patient education and communication in dentistry. Cureus.

[12] Kirchner GJ, Kim RY, Weddle JB, Bible JE (2023). Can artificial intelligence improve the readability of patient education materials?. Clin Orthop Relat Res.

[13] Jayakumar P, Moore MG, Furlough KA (2021). Comparison of an artificial intelligence-enabled patient decision aid vs educational material on decision quality, shared decision-making, patient experience, and functional outcomes in adults with knee osteoarthritis. JAMA Netw Open.

[14] Oliveira JA, Eskandar K, Kar E, de Oliveira FR, Filho AL (2024). Understanding AI’s role in endometriosis patient education and evaluating its information and accuracy: systematic review. JMIR AI.

[15] Sallam M (2023). ChatGPT utility in healthcare education, research, and practice: Systematic review on the promising perspectives and valid concerns. Healthcare (Basel).

[16] Das N, Panjabi M (2011). Plagiarism: why is it such a big issue for medical writers?. Perspect Clin Res.

[17] Helgesson G, Eriksson S (2015). Plagiarism in research. Med Health Care Philos.

[18] Montoya A, Llopis N, Gilaberte I (2011). Validation of the translation of an instrument to measure reliability of written information on treatment choices: a study on attention deficit/hyperactivity disorder (ADHD). Educ Health (Abingdon).

[19] Pal S, Bhattacharya M, Islam MA, Chakraborty C (2024). AI-enabled ChatGPT or LLM: a new algorithm is required for plagiarism-free scientific writing. Int J Surg.

[20] Banasiak NC, Meadows-Oliver M (2017). Evaluating asthma websites using the Brief DISCERN instrument. J Asthma Allergy.

[21] Kacer EO (2025). Evaluating AI-based breastfeeding chatbots: quality, readability, and reliability analysis. PLoS One.

